# Relationship between body composition and left ventricular geometry using three dimensional cardiovascular magnetic resonance

**DOI:** 10.1186/s12968-016-0251-4

**Published:** 2016-05-31

**Authors:** Ben Corden, Antonio de Marvao, Timothy J. Dawes, Wenzhe Shi, Daniel Rueckert, Stuart A. Cook, Declan P. O’Regan

**Affiliations:** Medical Research Council Clinical Sciences Centre, Faculty of Medicine, Imperial College London, Hammersmith Hospital Campus, London, UK; Department of Computing, Imperial College London, South Kensington Campus, London, UK; National Heart Centre Singapore, Singapore and Duke-NUS Graduate Medical School, Singapore, Singapore

**Keywords:** Obesity, Body composition, Cardiovascular magnetic resonance, Cardiac remodelling, Cardiac atlas

## Abstract

**Background:**

Although obesity is associated with alterations in left ventricular (LV) mass and volume which are of prognostic significance, widely differing patterns of remodelling have been attributed to adiposity. Our aim was to define the relationship between body composition and LV geometry using three-dimensional cardiovascular magnetic resonance.

**Methods:**

In an observational study 1530 volunteers (55 % female, mean age 41.3 years) without known cardiovascular disease underwent investigation including breath-hold high spatial resolution 3D cines. Atlas-based segmentation and co-registration was used to create a statistical model of wall thickness (WT) and relative wall thickness (RWT) throughout the LV. The relationship between bio-impedence body composition and LV geometry was assessed using 3D regression models adjusted for age, systolic blood pressure (BP), gender, race and height, with correction to control the false discovery rate.

**Results:**

LV mass was positively associated with fat mass in women but not in men (LV mass: women β = 0.11, *p* < 0.0001; men β = −0.01, *p* = 0.82). The 3D models revealed that in males fat mass was strongly associated with a concentric increase in relative wall thickness (RWT) throughout most of the LV (β = 0.37, significant area = 96 %) and a reduced mid-ventricular cavity (β = −0.22, significant area = 91 %). In women the regional concentric hypertrophic association was weaker, and the basal lateral wall showed an inverse relationship between RWT and fat mass (β = −0.11, significant area = 4.8 %).

**Conclusions:**

In an adult population without known cardiovascular disease increasing body fat is predominately associated with asymmetric concentric hypertrophy independent of systolic BP, with women demonstrating greater cavity dilatation than men. Conventional mass and volume measurements underestimate the impact of body composition on LV structure due to anatomic variation in remodelling.

**Electronic supplementary material:**

The online version of this article (doi:10.1186/s12968-016-0251-4) contains supplementary material, which is available to authorized users.

## Background

More than a third of adults are obese and obesity-related conditions are some of the leading causes of preventable death [1]. The increased incidence of cardiovascular disease is driven not only by diabetes and hypertension but also by cellular myocardial injury, left ventricular (LV) hypertrophy and diastolic dysfunction [2, 3]. Adiposity is thought to influence these adaptations via both haemodynamic and metabolic effects [4] with the cumulative exposure to obesity being a major predisposing factor [5]. As LV hypertrophy is independently associated with all-cause morbidity and mortality [6], the interaction between body composition and cardiac structure is critical for understanding the influence of this modifiable risk factor on cardiovascular health [7, 8].

The traditional view held that obesity was associated with eccentric LV hypertrophy due to the effect of obesity-related volume overload [9], however there is wide variation in the observed structural adaptations to obesity [10], with concentric hypertrophy, concentric remodelling, and eccentric hypertrophy described in different cohorts [11, 12]. Gender-differences have also been recognised as males develop more prognostically-adverse adaptations of LV geometry to obesity [13]. Three dimensional modelling of LV shape using high-spatial resolution cardiovascular magnetic resonance (CMR) offers a new approach for accurate quantitative assessment of whole-heart geometry in large populations [14, 15]. In this study we applied these methods to determine the relationship between body composition and LV structure in healthy adults.

## Methods

### Subjects

Between February 2011 and July 2015 a total of 1530 adult volunteers (55 % female, age range 18–81 years, mean ± standard deviation 41.3 ± 13.0 years) were prospectively recruited via advertisement to a single-site cross-sectional study (UK Digital Heart Project). The relationship between systolic blood pressure (BP) and LV geometry in this cohort has been previously reported [15]. We excluded participants at screening who had known cardiovascular disease or were being treated for hypertension, diabetes or hypercholesterolaemia. Standard safety contraindications to CMR were applied. Female subjects were excluded if they were pregnant or breastfeeding. All subjects provided written informed consent for participation in the study, which was approved by the National Research Ethics Service (09/H0707/69).

### Study protocol

CMR was performed on a 1.5 T Philips Achieva system (Best, Netherlands). The maximum gradient strength was 33 mT/m and the maximum slew rate 160 mT/m/ms. A 32 element cardiac phased-array coil was used for signal reception. Scout images were obtained and used to plan 2D cine balanced steady-state free precession (b-SSFP) images in the left ventricular short axis (LVSA) plane from base to apex using the following parameters: repetition time/echo time, 3.0 ms/1.5 ms; flip angle, 60°; bandwidth, 1250 Hz/pixel; acquired pixel size, 2.0 × 2.2 mm; section thickness 8 mm with a 2 mm gap; reconstructed voxel size, 1.2 × 1.2 × 8 mm; number of sections, 10–12; cardiac phases, 30. A single breath-hold 3D LVSA b-SSFP sequence was acquired in the same orientation using the following parameters: 3.0 ms/1.5 ms; flip angle, 50°; bandwidth, 1250 Hz/pixel; pixel size 2.0 × 2.0 mm; section thickness, 2 mm overlapping; reconstructed voxel size, 1.2 × 1.2 × 2 mm; number of sections, 50–60; cardiac phases, 20; sensitivity encoding (SENSE) factor 2.0 anterior-posterior and 2.0 right-left direction [14]. Images were curated on an open-source image database (MRIdb, Imperial College London, UK) [16].

Left ventricular end-diastolic volume (LVEDV), left ventricular mass (LVM) and stroke volume (SV) were measured from the cine images using CMRtools (Cardiovascular Imaging Solutions, London, UK) according to international guidelines [17]. Cardiac output (CO) was derived by multiplying SV by heart rate (HR). Concentricity was calculated as LVM/LVEDV.

### Three-dimensional analysis

All image processing was performed in Matlab (Mathworks, Natick, MA) using a biventricular atlas of cardiac structure and function [18]. Segmentation of the 3D cine images was performed using an algorithm which searches for correspondences between small cubic regions, or patches, in the image to be segmented and a database of labelled atlases while also matching global shape features [19]. The process was initialised by a reader placing six pre-defined anatomical landmarks on the target images which were also defined on each labelled atlas (left ventricular apex, mitral annulus and lateral wall; the RV freewall; and the superior and inferior RV insertion points). The final segmentations were co-registered to a mean 3D template of the healthy volunteers to make the data anatomically consistent between each subject and provide a smooth interpolation of cardiac shape. Wall thickness (WT) was calculated at end-diastole and was measured as the distance between the endocardial and epicardial surfaces perpendicular to the midwall plane. Relative wall thickness (RWT) was determined by adjusting the WT measurements for variations in LV volume using a scale transformation of the 3D model. Variation in the position of the endocardial and epicardial surfaces was determined relative to an average cardiac shape [15]. A regional concentric hypertrophic association was indicated by positive regression coefficients for both WT and RWT.

### Body composition and blood pressure

All measurements were performed by cardiology nurses at the study centre. Height and weight were measured without shoes while wearing scrubs. Total body fat mass and lean mass were measured using bioelectrical impedance (InBody 230, BioSpace, Los Angeles, CA) [20]. Blood pressure was measured after 5 min rest in accordance with European Society of Hypertension guidelines [21] using a calibrated oscillometric device (Omron M7, Omron Corporation, Kyoto, Japan) that has been validated in both normal [22] and obese populations [23]. The first of three measures was discarded and the mean of the second two values was recorded.

### Statistical analysis

Statistical analysis was carried out using RStudio Server version 0.98 (Boston, MA). Data are reported as mean ± standard deviation. The associations between body composition and LV mass, LV EDV, concentricity, SV and HR were assessed in separate multiple linear regression models, with adjustment for age, systolic BP, gender, race and height. Race was dummy-coded with the largest group, Caucasian, as the reference. Interaction terms for gender and body composition were included and, where there was a significant interaction, this was explored further with separate regression models for men and women. The associations between body composition and 3D phenotypic parameters (WT, RWT, endocardial shape and epicardial shape) were assessed using regression models adjusted for age, systolic BP, gender, race and height, with correction to control the false discovery rate [24]. Contiguous regional effects in the left ventricle were identified where the association between variables was significant (*p* < 0.05) and are reported as the mean of the standardized β coefficients within that area. Comparison between groups and regression models was performed using analysis of variance, corrected for covariates.

## Results

### Study population characteristics

Summary statistics for the main variables are shown in Table 1. The BMI range of the cohort was from 12.7 to 41.9 kg/m^2^.

**Table 1:** Baseline characteristics stratified by gender

	Women	Men
	*n = 839*	*n = 691*
Age (years)	41.5 ± 13.4	40.9 ± 12.5
Race / Ethnicity:		
Caucasian	626 (74.6 %)	518 (75.0 %)
Asian	97 (11.6 %)	97 (14.0 %)
African	65 (7.7 %)	39 (5.6 %)
Other	51 (6.1 %)	37 (5.4 %)
BMI (kg/m^2^)	24.3 ± 4.2	25.1 ± 3.3
Underweight (BMI <18.5)	22 (2.6 %)	8 (1.2 %)
Normal weight (18.5 ≤ BMI < 25)	528 (62.9 %)	359 (52.0 %)
Overweight (25 ≤ BMI < 30)	200 (23.8 %)	270 (39.1 %)
Obese (BMI ≥ 30)	89 (10.6 %)	54 (7.8 %)
Lean Mass (kg)	44.9 ± 6.0	62.6 ± 8.5
BMI <18.5	36.9 ± 4.5	48.5 ± 4.4
18.5 ≤ BMI < 25	44.1 ± 5.2	59.8 ± 7.5
25 ≤ BMI < 30	46.4 ± 6.1	65.0 ± 8.5
BMI ≥ 30	50.5 ± 6.9	70.9 ± 8.1
Fat Mass (kg)	20.3 ± 8.5	15.9 ± 7.3
BMI <18.5	9.0 ± 2.3	5.0 ± 2.3
18.5 ≤ BMI < 25	16.1 ± 4.0	11.5 ± 3.9
25 ≤ BMI < 30	26.2 ± 5.1	18.9 ± 6.0
BMI ≥ 30	36.4 ± 8.0	29.8 ± 6.1
Systolic BP (mmHg)	114.0 ± 13.5	123.5 ± 12.9
BMI <18.5	103.7 ± 9.3	106.8 ± 8.3
18.5 ≤ BMI < 25	111.8 ± 12.3	122.9 ± 13.8
25 ≤ BMI < 30	118.7 ± 14.1	125.2 ± 12.5
BMI ≥ 30	123.9 ± 14.9	128.7 ± 15.9
Diastolic BP (mmHg)	77.2 ± 9.3	79.8 ± 9.5
BMI <18.5	73.5 ± 8.7	72.5 ± 7.9
18.5 ≤ BMI < 25	75.6 ± 8.9	78.6 ± 8.9
25 ≤ BMI < 30	80.6 ± 9.3	81.6 ± 9.1
BMI ≥ 30	83.4 ± 10.2	85.5 ± 12.1
LV Mass (g)	99.3 ± 19.3	79.8 ± 9.5
LV EDV (mls)	128.9 ± 21.6	165.4 ± 31.1
LV SV (mls)	85.8 ± 14.0	105.6 ± 19.4
LV EF (%)	66.8 ± 5.0	64.1 ± 5.4
Cardiac Output (L/min)	5.6 ± 1.2	6.6 ± 1.5

Data are expressed as mean ± SD. *BMI* indicates body mass index, *BP* blood pressure, *EDV* end diastolic volume, *SV* stroke volume, and *EF* ejection fraction

### Relationships between fat mass and LV geometry

Summaries of the regression models using the conventional CMR data are shown in Table 2. Complete multiple linear regression models are provided in the Additional file 1: Table S1 and split by gender in Additional file 2: Table S2.

**Table 2:** Summary of the multiple linear regression models, split by gender

	Men			Women			
	B	β	*P*	B	β	*p*	*p* for interaction
LV mass (g)							
Lean Mass	1.91	0.53	<.0001	1.65	0.51	<.0001	.005
Fat Mass	−0.03	−0.01	.82	0.26	0.11	<.0001	.02
LV EDV (ml)							
Lean Mass	1.80	0.49	<.0001	1.56	0.43	<.0001	.11
Fat Mass	−0.29	−0.07	.02	0.34	0.13	<.0001	<.0001
Concentricity (LV mass/volume)							
Lean Mass	0.002	0.11	.03	0.004	0.19	<.0001	.65
Fat Mass	0.002	0.09	.03	−6×10^−6^	−0.0004	.99	.03
Stroke Volume (ml)							
Lean Mass	1.01	0.44	<.0001	1.07	0.46	<.0001	.86
Fat Mass	−0.1	−0.04	.23	0.28	0.17	<.0001	<.0001
Heart Rate (bpm)							
Lean Mass	−0.26	−0.20	.0003	−0.15	−0.10	.04	.48
Fat Mass	0.26	0.17	<.0001	0.09	0.08	.04	.04
Cardiac Output (L/min)							
Lean Mass	0.03	0.18	.0002	0.06	0.29	<.0001	.19
Fat Mass	0.02	0.10	.006	0.03	0.20	<.0001	.09

Models are adjusted for age, race, systolic BP and height. Lean mass and fat mass are in kg

B gives the estimate of the beta-values in the regression equations, such that for each 1 kg increase in fat mass or lean mass the given predictor variable (e.g. LV mass) changes by B amount (if other variables in the model are held constant)

β gives standardised beta-values, such that for each 1 standard-deviation increase in lean mass or fat mass, the given predictor variable changes by β standard-deviations (if other variables in the model are held constant)

*R* ^*2*^ for LV mass models: men = 0.42, women = 0.43. *R* ^*2*^ for LV EDV models: men = 0.47, women = 0.45. *R* ^*2*^ for concentricity models: men = 0.10, women = 0.10. *R* ^*2*^ for stroke volume models: men = 0.42, women = 0.45. *R* ^*2*^ for heart rate models: men = 0.06, women = 0.02. *R* ^*2*^ for cardiac output models: men = 0.24, women = 0.29. *BP* indicates blood pressure, *LV* left ventricle and *EDV* end diastolic volume

#### LV wall thickness, mass and volume

**Fig. 1:**
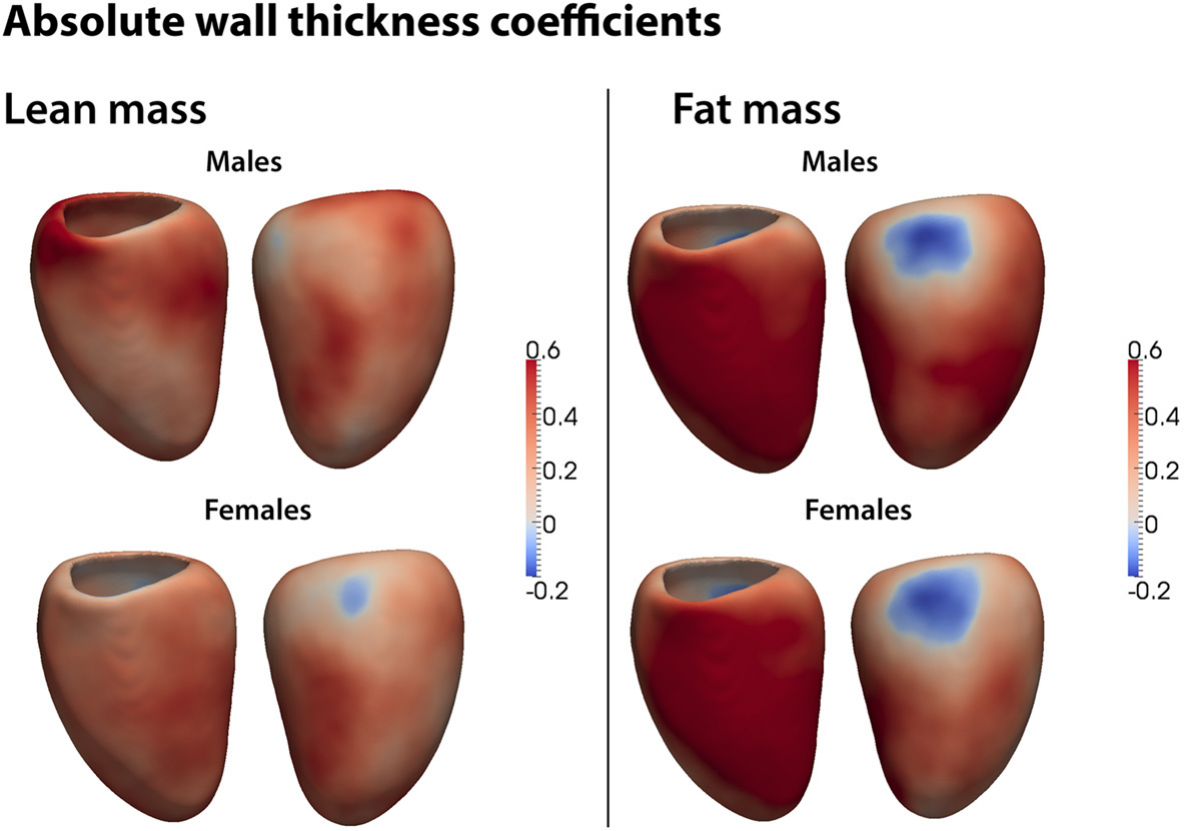
Three dimensional regression models of the association between absolute wall thickness (WT) and body composition in the left ventricle. The effects of lean and fat mass are shown separately for men and women. Myocardium shown in red indicates a positive relationship between WT and either fat or lean mass, and myocardium in blue a negative relationship. Anterior and lateral views of the left ventricle are shown

Global LV mass was weakly associated with fat mass in women but not in men (women β = 0.11, *p* < 0.0001; men β = −0.01, *p* = 0.82; p for interaction = 0.02). However, there were strong and distinct regional variations in the relationship between LV geometry and adiposity that were revealed by the 3D regression models. There was a regional hypertrophic relationship between fat mass and WT throughout most of the left ventricle in both sexes, but this relationship was stronger and more extensive in men, with women showing greater asymmetry (females β = 0.25, significant area = 94 %, males β = 0.31, significant area = 95 %) (Fig. 1). Adiposity was also associated with characteristic changes in the shape and volume of the left ventricle. In males, increasing fat mass was associated with a global reduction in endocardial volume but predominantly affecting the septal wall (β = −0.22, significant area = 91 %). By contrast, in females fat mass was also associated with an increase in endocardial volume at basal and mid-ventricular levels (females β =0.07, significant area = 22 %). These observations from the 3D regression models are reflected in the changes seen in global LV EDV, which showed a significant positive association with fat mass in women but a weakly negative association in men (women β = 0.13, *p* < .0001; men β = −0.07, *p* = .02; p for interaction < .0001). Females demonstrated a mild expansion of the epicardial surface in association with increasing fat mass (β = 0.08, significant area = 68 %), which was confined to the septum and lateral wall in males (β = 0.04, significant area = 28 %).

#### Concentricity: relative wall thickness and LV mass:volume ratio

**Fig. 2:**
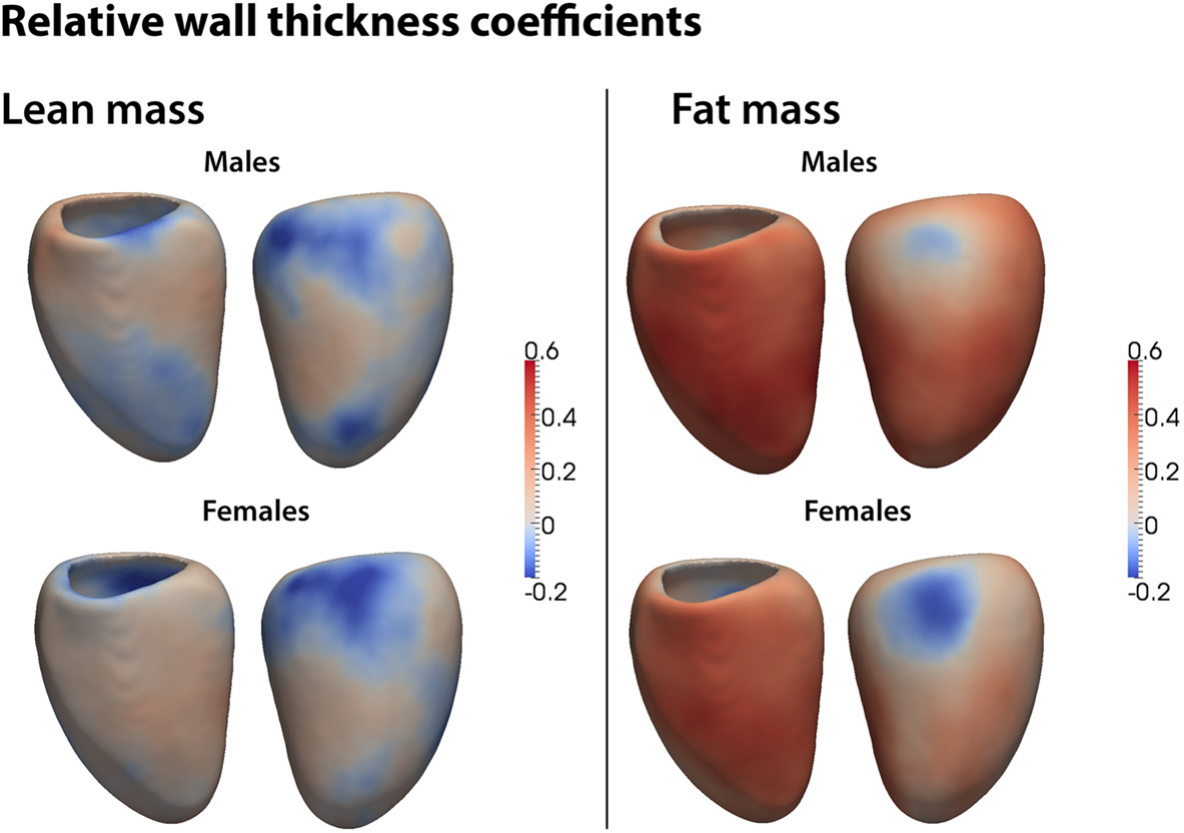
Three dimensional regression models of the association between relative wall thickness (RWT) and body composition in the left ventricle. The effects of lean and fat mass are shown separately for men and women. Myocardium shown in red indicates a positive relationship between RWT and either fat or lean mass, and myocardium in blue a negative relationship. Anterior and lateral views of the left ventricle are shown

The net effect of the regional changes in WT and myocardial shape were evident in the relationship between fat mass and both RWT and global concentricity in men and women (Fig. 2). In men, fat mass was associated with an increase in RWT throughout most of the left ventricle (β = 0.37, significant area = 96 %) consistent with a concentric hypertrophic pattern. In women fat mass was also associated with concentric hypertrophy throughout most of the left ventricle but the relationship was less strong and more asymmetrical than in men - including an area without hypertrophy in the basal lateral wall (β = −0.11, significant area = 4.8 %). Due to the regionality of remodelling the global LV mass:volume ratio only showed a weak positive association with adiposity in men but not in women (men β = 0.09, *p* = 0.03; women β = −0.0004, *p* = 0.99; p for interaction = 0.03).

### Relationships between lean mass and LV geometry

**Fig. 3:**
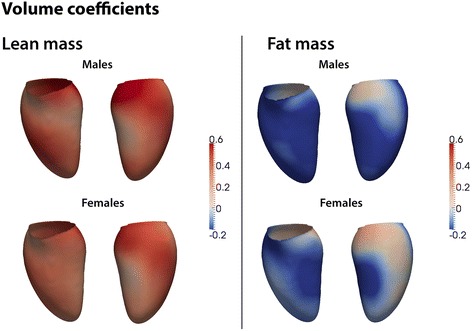
Three dimensional regression models of the association between left ventricular shape and body composition. The association between lean and fat mass with change in shape of the endocardial surface are shown separately for men and women. Positive coefficients (*red*) indicate an expansion in volume and a negative coefficient (*blue*) indicates a reduction in volume. Anterior and lateral views of the left ventricle are shown

Lean mass showed a strongly positive association with global LV mass for both genders, with higher coefficients in men (men: β = 0.58, *p* < 0.0001; women β = 0.55, *p* < 0.0001; p for interaction = .005). However, this masked a complex effect on regional LV geometry. In both sexes lean mass was associated with a generalised increase in WT with the strongest effect in the septum (females β = 0.21, significant area = 90 %, males β = 0.23, significant area = 96 %). This hypertrophic association with lean mass was linked with an expansion of both the endocardial and epicardial surfaces with an overall effect of a mild concentric increase in septal RWT (females β =0.10, significant area = 22.4 %; males β = 0.10, significant area = 14.7 %) (Fig. 3).

## Discussion

Increasing body fat is associated with changes in three dimensional LV geometry in otherwise healthy adults without known co-morbidities. The septum is most sensitive to changes in body composition even after adjusting for systolic BP. While increasing adiposity in both sexes is associated with a predominantly a concentric hypertrophic pattern, females show a more asymmetric adaptation with cavity dilatation. The relationship between LV geometry and body composition can be accurately demonstrated using 3D imaging with atlas-based modelling.

### Mechanisms contributing to cardiac remodelling in obesity

Early data from echocardiography pointed towards eccentric hypertrophy (LV dilatation and hypertrophy) in obesity due to a mildly volume-overload state [25]. In contrast, later CMR-derived cohort data indicated that obesity was associated with concentric LV remodelling without a change in ejection fraction [26]. However, this population had a high prevalence of hypertension, diabetes and hypercholesterolaemia so it was not possible to determine the independent effect of obesity on cardiac geometry. More recent CMR data in healthy adults, free of known cardiovascular disease, supported these findings and demonstrated that increasing BMI was strongly related to increasing LV mass independent of hypertension [13]. As well as fat mass leading to alterations in afterload and preload, LV geometry is also influenced by pro-inflammatory factors, glucotoxicity, lipotoxicity and leptin-resistance [27]. Measuring how the heart remodels in response to these complex haemodynamic and non-haemodynamic factors has relied on global indices of mass, volume and concentricity. However, it is recognised that assumptions of anatomic uniformity can be misleading as regional changes in wall thickness, radius of curvature and deformation are characteristically associated with altered loading conditions and intrinsic myocardial disease [28, 29]. In this study we used 3D imaging to determine how the left ventricle adapts to altered body composition without imposing geometric assumptions and acquired at near-isotropic resolution.

### Relationship between cardiac structure and body composition

Using 3D modelling we found that body fat mass was positively associated with WT throughout most of the myocardium but the relationship was strongest in the septal wall. The increase in WT was associated with a smaller cavity making the relationship between body fat and RWT predominantly one of concentric hypertrophy. Structural adaptations were not uniform and the basal lateral wall demonstrated a negative association between fat mass and RWT due to regional changes in LV shape. A consequence of this asymmetry was that the association between fat mass and RWT was stronger in some regions of the heart than was evident from global LV mass and volume measurements. The underlying mechanisms for anatomic variation in the hypertrophic response are not fully understood but the rate of increase in regional stress relative to pressure is greatest in the septum and therefore concentric adaptation to altered loading conditions may preferentially develop in an asymmetric pattern [30]. Lean and fat mass tend to increase together in obesity [31], and we found that while fat mass was strongly associated with concentric hypertrophy lean mass was associated with eccentric hypertrophy due to an expansion in endocardial shape. It is also possible that deficiency of fat-free mass could mediate the relationship between LV mass index and central fat distribution in obese subjects [32]. Our data show that LV mass and volume do not fully reflect the regional adaptations of the ventricle to changes in body composition.

### Gender differences in remodelling

It has been previously reported that in response to increasing body fat males demonstrate a progressive concentric hypertrophic process without LV cavity dilatation, while the pattern in women exhibits aspects of both concentric and eccentric remodelling [13]. Our data show that although concentric hypertrophy is predominant in both sexes women show a stronger association between fat mass and a regional increase in cardiac volume. A potential mechanism relates to gender differences in how body fat is stored and the consequent haemodynamic effect. A visceral distribution of body fat, more common in males [33], is associated with concentric LV remodelling, whereas a peripheral distribution of body fat, more common in females, is associated with eccentric LV remodelling and a higher CO [34]. These morphological adaptations are independent of systolic BP and suggest that changes in both pre-load and afterload are influential in determining local patterns of remodelling.

### Limitations

We used a bioimpedence device that obtains a direct impedance measurement using an 8-point tactile electrode and multi-frequency analysis which does not depend on empirical statistical models. Although this specific equipment is accurate and reproducible compared to dual-energy X-ray absorptiometry it may not be equally accurate in all body types and does not define the internal distribution of body fat [20, 34, 35]. Our anatomic measurements were adjusted for both height and gender to avoid obtaining biased estimates between men and women, however we did not apply allometric scaling or non-linear regression models to our 3D data [36]. We examined the association between variables using regression models and did not apply thresholds for hypertrophy or dilatation in this healthy population. Ours is the largest reported study of the relationship between body composition and cardiac geometry in healthy adults, but its cross-sectional design meant that we could not establish causal relationships or determine longitudinal trends in remodelling [37]. Due to the constraints of CMR we could not study subjects with severe obesity and this may have introduced a selection bias.

## Conclusions

In an adult population without known cardiovascular disease increasing body fat is predominately associated with asymmetric concentric hypertrophy independent of systolic BP, with women demonstrating greater cavity dilatation than men. Conventional mass and volume measurements underestimate the impact of body composition on LV structure due to anatomic variation in remodelling.

## Abbreviations

CMR, Cardiovascular magnetic resonance; EDV, End-diastolic volume; LV, Left ventricular; RV, Right ventricular; RWT, Relative wall thickness; WT, Wall thickness

## Additional files

Additional file 1: Table S1. Complete multiple linear regression models. (DOCX 17 kb) Additional file 2: Table S2. Multiple linear regression models split by gender. (DOCX 18 kb)
