# Supplementary material for: Relationship between body composition and left ventricular geometry using three dimensional cardiovascular magnetic resonance
Source: J Cardiovasc Magn Reson. 2016 May 31;18:32. doi: 10.1186/s12968-016-0251-4 (PMC4888671; doi:10.1186/s12968-016-0251-4)
Supplement: Additional file 1: Table S1. — Complete multiple linear regression models. (DOCX 17 kb) [file 12968_2016_251_MOESM1_ESM.docx]

**Supplementary table 1.** Complete multiple linear regression models.

|  |  | Standardised β | *p* |  |  |
| --- | --- | --- | --- | --- | --- |
| LV mass | |  |  |  |  |
|  | Age | -0.14 | <.0001 |  |  |
|  | Gender | -0.05 | 0.69 |  |  |
|  | Race: C v AF | 0.04 | .005 |  |  |
|  | Race: C v Asian | -0.11 | <.0001 |  |  |
|  | Race: C v Other | -0.04 | .01 |  |  |
|  | Systolic BP | 0.15 | <.0001 |  |  |
|  | Height | -0.03 | 0.24 |  |  |
|  | Lean Mass | 0.53 | <.0001 |  |  |
|  | Fat Mass | 0.06 | .003 |  |  |
|  | Gender X Lean Mass | 0.37 | .005 |  |  |
|  | Gender X Fat Mass | -0.08 | .02 |  |  |
| LV EDV | |  |  |  | |
|  | Age | -0.20 | <.0001 |  |  |
|  | Gender | -0.007 | .95 |  |  |
|  | Race: C v AF | -0.07 | <.0001 |  |  |
|  | Race: C v Asian | -0.15 | <.0001 |  |  |
|  | Race: C v Other | -0.03 | .06 |  |  |
|  | Systolic BP | 0.07 | .0002 |  |  |
|  | Height | 0.12 | .0001 |  |  |
|  | Lean Mass | 0.55 | <.0001 |  |  |
|  | Fat Mass | 0.09 | <.0001 |  |  |
|  | Gender X Lean Mass | 0.23 | .11 |  |  |
|  | Gender X Fat Mass | -0.19 | <.0001 |  |  |
| Concentricity | |  |  |  | |
|  | Age | 0.03 | .24 |  |  |
|  | Gender | 0.27 | .12 |  |  |
|  | Race: C v AF | 0.14 | <.0001 |  |  |
|  | Race: C v Asian | -0.003 | .90 |  |  |
|  | Race: C v Other | -0.02 | .41 |  |  |
|  | Systolic BP | 0.16 | <.0001 |  |  |
|  | Height | -0.17 | <.0001 |  |  |
|  | Lean Mass | 0.23 | .001 |  |  |
|  | Fat Mass | -0.007 | .82 |  |  |
|  | Gender X Lean Mass | -0.09 | .65 |  |  |
|  | Gender X Fat Mass | 0.12 | .03 |  |  |
| Stroke Volume | |  |  |  |  |
|  | Age | -0.15 | <.0001 |  |  |
|  | Gender | 0.08 | .52 |  |  |
|  | Race: C v AF | -0.08 | <.0001 |  |  |
|  | Race: C v Asian | -0.14 | <.0001 |  |  |
|  | Race: C v Other | -0.04 | .01 |  |  |
|  | Systolic BP | 0.12 | <.0001 |  |  |
|  | Height | 0.14 | <.0001 |  |  |
|  | Lean Mass | 0.60 | <.0001 |  |  |
|  | Fat Mass | 0.13 | <.0001 |  |  |
|  | Gender X Lean Mass | 0.03 | 0.86 |  |  |
|  | Gender X Fat Mass | -0.20 | <.0001 |  |  |
| Heart Rate | | | |  |  |
|  | Age | -0.08 | .004 |  |  |
|  | Gender | 0.02 | .91 |  |  |
|  | Race: C v AF | -0.03 | .18 |  |  |
|  | Race: C v Asian | 0.01 | .76 |  |  |
|  | Race: C v Other | -0.01 | .83 |  |  |
|  | Systolic BP | 0.13 | <.0001 |  |  |
|  | Height | 0.07 | .17 |  |  |
|  | Lean Mass | -0.20 | .01 |  |  |
|  | Fat Mass | 0.08 | .02 |  |  |
|  | Gender X Lean Mass | -0.16 | .48 |  |  |
|  | Gender X Fat Mass | 0.12 | .04 |  |  |
| Cardiac Output | | | |  |  |
|  | Age | -0.19 | <.0001 |  |  |
|  | Gender | 0.21 | .20 |  |  |
|  | Race: C v AF | -0.09 | <.0001 |  |  |
|  | Race: C v Asian | -0.11 | <.0001 |  |  |
|  | Race: C v Other | -0.04 | .06 |  |  |
|  | Systolic BP | 0.19 | <.0001 |  |  |
|  | Height | 0.19 | <.0001 |  |  |
|  | Lean Mass | 0.40 | <.0001 |  |  |
|  | Fat Mass | 0.17 | <.0001 |  |  |
|  | Gender X Lean Mass | -0.25 | .19 |  |  |
|  | Gender X Fat Mass | -0.08 | .09 |  |  |

*R^2^* for LV mass model: 0.68, *R^2^* for LV EDV model: 0.63, *R^2^* for concentricity model: 0.24, *R^2^* for stroke volume model: 0.58, *R^2^* for heart rate model: 0.05

BP indicates blood pressure; LV, left ventricle; EDV, end diastolic volume.
