# Supplementary material for: Relationship between body composition and left ventricular geometry using three dimensional cardiovascular magnetic resonance
Source: J Cardiovasc Magn Reson. 2016 May 31;18:32. doi: 10.1186/s12968-016-0251-4 (PMC4888671; doi:10.1186/s12968-016-0251-4)
Supplement: Additional file 2: Table S2. — Multiple linear regression models split by gender. (DOCX 18 kb) [file 12968_2016_251_MOESM2_ESM.docx]

**Supplementary table 2.** Multiple linear regression models split by gender.

|  |  | Men | | Women | |
| --- | --- | --- | --- | --- | --- |
|  |  | Standardised β | *p* | Standardised β | *p* |
| LV mass | |  |  |  |  |
|  | Age | -0.17 | <.0001 | -0.21 | <.0001 |
|  | Race: C v AF | 0.05 | .13 | 0.08 | .004 |
|  | Race: C v Asian | -0.20 | <.0001 | -0.09 | .002 |
|  | Race: C v Other | -0.05 | .08 | -0.05 | .08 |
|  | Systolic BP | 0.19 | <.0001 | 0.19 | <.0001 |
|  | Height | -0.05 | .27 | -0.01 | .70 |
|  | Lean Mass | 0.53 | <.0001 | 0.51 | <.0001 |
|  | Fat Mass | -0.01 | .82 | 0.11 | <.0001 |
| LV EDV | |  |  |  |  |
|  | Age | -0.21 | <.0001 | -0.27 | <.0001 |
|  | Race: C v AF | -0.11 | <.0001 | -0.06 | .03 |
|  | Race: C v Asian | -0.20 | <.0001 | -0.15 | <.0001 |
|  | Race: C v Other | -0.02 | .55 | -0.06 | .03 |
|  | Systolic BP | 0.07 | .01 | 0.08 | .01 |
|  | Height | 0.07 | .08 | 0.14 | <.0001 |
|  | Lean Mass | 0.49 | <.0001 | 0.43 | <.0001 |
|  | Fat Mass | -0.07 | .02 | 0.13 | <.0001 |
| Concentricity – LV mass / LV EDV | | | | | |
|  | Age | 0.04 | .30 | 0.03 | .50 |
|  | Race: C v AF | 0.17 | <.0001 | 0.15 | <.0001 |
|  | Race: C v Asian | -0.04 | .26 | 0.05 | .20 |
|  | Race: C v Other | -0.04 | .25 | 0.001 | .99 |
|  | Systolic BP | 0.17 | <.0001 | 0.16 | <.0001 |
|  | Height | -0.11 | .04 | -0.16 | .0002 |
|  | Lean Mass | 0.11 | .03 | 0.19 | <.0001 |
|  | Fat Mass | 0.09 | .03 | -0.0004 | .99 |
| Stroke volume | |  |  |  |  |
|  | Age | -0.17 | <.0001 | -0.19 | <.0001 |
|  | Race: C v AF | -0.10 | .0005 | -0.10 | <.0001 |
|  | Race: C v Asian | -0.18 | <.0001 | -0.13 | <.0001 |
|  | Race: C v Other | -0.02 | .42 | -0.08 | .004 |
|  | Systolic BP | 0.12 | <.0001 | 0.14 | <.0001 |
|  | Height | 0.11 | .01 | 0.13 | .0002 |
|  | Lean Mass | 0.44 | <.0001 | 0.46 | <.0001 |
|  | Fat Mass | -0.04 | .23 | 0.17 | <.0001 |
| Heart Rate | | | |  |  |
|  | Age | -0.11 | .005 | -0.06 | .16 |
|  | Race: C v AF | -0.02 | .64 | -0.05 | .17 |
|  | Race: C v Asian | 0.004 | .92 | 0.01 | .80 |
|  | Race: C v Other | 0.009 | .81 | -0.02 | .66 |
|  | Systolic BP | 0.11 | .005 | 0.13 | .001 |
|  | Height | 0.07 | .17 | 0.03 | .54 |
|  | Lean Mass | -0.20 | .0003 | -0.10 | .04 |
|  | Fat Mass | 0.17 | <.0001 | 0.08 | .04 |
| Cardiac Output | | | | | |
|  | Age | -0.22 | <.0001 | -0.19 | <.001 |
|  | Race: C v AF | -0.08 | .01 | -0.11 | .0004 |
|  | Race: C v Asian | -0.14 | <.0001 | -0.10 | .001 |
|  | Race: C v Other | -0.01 | .74 | -0.07 | .02 |
|  | Systolic BP | 0.18 | <.0001 | 0.20 | <.0001 |
|  | Height | 0.17 | .0006 | 0.13 | .001 |
|  | Lean Mass | 0.18 | .0002 | 0.29 | <.0001 |
|  | Fat Mass | 0.10 | .006 | 0.20 | <.0001 |

*R^2^* for LV mass models: men = 0.42, women = 0.43. *R^2^* for LV EDV models: men = 0.47 , women = 0.45 *R^2^* for concentricity models: men = 0.10, women = 0.10. *R^2^* for stroke volume models: men = 0.42, women = 0.45, *R^2^* for heart rate models: men = 0.06, women = 0.02, *R^2^* for cardiac output models: men = 0.24, women = 0.29. BP indicates blood pressure; LV, left ventricle and EDV, end diastolic volume. Concentricity = LV mass / LV EDV.
